# Vitamin D serum level is associated with Child–Pugh score and metabolic enzyme imbalances, but not viral load in chronic hepatitis B patients

**DOI:** 10.1097/MD.0000000000003926

**Published:** 2016-07-08

**Authors:** Xin-yan Zhao, Jia Li, Jing-han Wang, Sohail Habib, Wei Wei, Shu-jie Sun, Henry W. Strobel, Ji-dong Jia

**Affiliations:** a Liver Research Center, Beijing Friendship Hospital, Capital Medical University, Beijing Key Laboratory on Translational Medicine on Cirrhosis, National Clinical Research Center for Digestive Diseases, Beijing; b Tianjin Institute of Hepatology, Tianjin Second People's Hospital, Tianjin; c Clinical Laboratory, the Second Hospital of Dalian Medical University, Dalian, Liaoning; d International School, Capital Medical University, Beijing, People's Republic of China; e Department of Biochemistry & Molecular Biology, University of Texas Medical School, Houston, TX.

**Keywords:** 25-hydroxyvitamin D3, chronic hepatitis B, cirrhosis, hydroxylase, liver dysfunction

## Abstract

Vitamin D deficiency is common in patients with chronic liver diseases. However, vitamin D status in persons with chronic hepatitis B virus (HBV) infection is not consistently reported. Specifically, the impact of liver dysfunction on vitamin D status has not been well addressed.

We recruited a group of patients (n = 345) with chronic hepatitis B (n = 115), hepatitis B related cirrhosis (n = 115), and age- and gender-matched healthy controls (n = 115). Serum 25-hydroxyvitamin D3 [25(OH)D3], its related metabolic enzymes, intact parathyroid hormone were measured. Calcium, magnesium, and phosphorus were obtained from medical record.

Serum 25(OH)D3 levels in chronic hepatitis B patients (7.83 ± 3.47 ng/mL) were significantly lower than that in healthy controls (9.76 ± 4.36 ng/mL, *P* < 0.001), but significantly higher than that in hepatitis B-related cirrhotic patients (5.21 ± 3.67 ng/mL, *P* < 0.001). Furthermore, 25(OH)D3 decreased stepwise with higher Child–Pugh classification. However, there were no significant differences in 25(OH)D3 levels between (1) hepatitis B e antigen (HBeAg +) and HBeAg(–) persons, or (2) among persons with different HBV viral load, or (3) between treatment naïve and patients on antiviral therapy. Multiple logistic regression analyses confirmed that higher Child–Pugh score was independently associated with 25(OH)D3 deficiency (<10 ng/mL) with an odds ratio of 1.20 (confidence interval 1.03–1.39, *P* = 0.016). Levels of cytochrome P450 (CYP) 27A1 were significantly decreased, whereas levels of CYP24A1 were significantly elevated in cirrhotic patients.

These results suggest that decreasing vitamin D levels are likely to be a result, rather than a cause, of liver dysfunction and irrespective of HBV viral load. Reduction in 25(OH)D3 levels is possibly due to downregulation of the synthetic hydroxylase CYP27A1 and concurrent upregulation of degrading CYP24A1 in patients with liver cirrhosis.

## 1 Introduction

Vitamin D_3_ photochemically synthesized in the skin from 7-dehydrocholesterol has numerous activities in addition to its key role in maintaining bone homeostasis.^[1]^ Vitamin D insufficiency or deficiency has been well established in cholestatic liver diseases such as primary biliary cirrhosis, with malabsorption of fat soluble vitamins being the major cause of these phenomena.^[2]^ Recent studies have demonstrated the association of vitamin D deficiency with noncholestatic liver diseases such as chronic hepatitis C (CHC),^[3,4]^ alcoholic,^[5]^ or nonalcoholic fatty liver diseases.^[6–8]^ Furthermore, low vitamin D serum concentration is reported to be an independent factor of poor prognosis in alcoholic liver diseases^[5]^ and suboptimal antiviral response in patients with CHC.^[9,10]^

However, studies on vitamin D status in chronic HBV-infected persons have generated inconsistent results. Farnik et al^[11]^ and Wong et al^[12]^ reported that vitamin D deficiency in chronic hepatitis B (CHB) patients was associated with high HBV replication or adverse outcomes, respectively. In contrast, others showed that vitamin D deficiency was not associated with high viral load or poor prognosis in CHB.^[13,14]^ Physiologically, liver plays a key role in the activation of vitamin D since the precursor of vitamin D is hydroxylated to its main circulating form, 25(OH)D3 in the liver. This conversion process is mediated by cytochrome P450 (CYP) isoforms in the liver: microsomal CYP2R1 and mitochondrial CYP27A1. Therefore, one possible mechanism of vitamin D insufficiency or deficiency is due to dysfunction of the liver. Unfortunately, patients enrolled in these studies were mainly noncirrhotic and the association between vitamin D and liver dysfunction were not carefully evaluated.

To clarify this issue, we investigated the serum vitamin D levels in persons with CHB, hepatitis B-related cirrhosis, and in healthy controls.

## 2 Patients and methods

### 2.1 Patients

Patients (n = 940) were recruited at Beijing Friendship Hospital, Capital Medical University. In the CHB infection group, patients were required to display positivity of hepatitis B surface antigen (HBsAg) for more than 6 months with persistent or intermittent elevation in alanine transaminase before antiviral therapy.^[15–17]^ In the HBV-related cirrhosis group, patients were required to exhibit at least one of the following criteria: (1) histological presence of pseudo lobule; (2) presence of esophageal varices by endoscopy, and albumin < 35.0 g/L or International Normalized Ratio > 1.3; or (3) liver stiffness measurement (by Fibroscan)  > 12.4 Kpa. Patients were excluded if they (1) had concomitant diseases such as alcoholic liver disease; (2) were coinfection with HCV, hepatitis delta, or human immunodeficiency virus; (3) had renal dysfunction; (4) had a malignancy; (5) used illegal drugs; (6) had coronary heart disease; or if they (7) had received vitamin D or calcium supplements, bisphosphonates, calcitonin, or hormone-replacement therapy.

During the same period of time, we also recruited 408 healthy volunteers without HBV infection from the Health Check Center, Beijing Friendship Hospital, Capital Medical University. Persons were included only if they had normal results on liver and kidney function tests, chest X-ray, Electrocardiogram, and liver ultrasound. We excluded persons with diseases that could affect vitamin D metabolism, such as osteoporosis, diabetes, and hypertension. All subjects were Chinese Han and resided in the northern region of China (latitude, 38°3′ N—39°54′ N).

For all participants, pertinent medical history along with viral markers (hepatitis B surface antigen, HBeAg, HBe antibody, and HBV-DNA), liver biochemistry, renal function, complete blood count, minerals, such as calcium, magnesium, phosphorus, coagulating factors, and image profiles were obtained from medical records. All blood samples were obtained at fasting status and serum stored at –80°C for subsequent use.

The study protocol was reviewed and approved by the ethical committees of Beijing Friendship Hospital, Capital Medical University.

### 2.2 Measurement of serum 25(OH)D3

Serum 25(OH) D_3_ levels were measured in 345 samples by High Performance Liquid Chromatography Tandem Mass Spectrometry method (HPLC-TMS) with HPLC (Agilent 1100 Series, Palo Alto, CA) and TMS of API 3000 (AB SCIEX Headquarters, Framingham, MA) at the Lawke Health Laboratory Center (Beijing, China; College of American Pathology Certification number: 7197649), as previously described.^[18,19]^

### 2.3 Measurement of serum intact parathyroid hormone

Intact parathyroid hormone (PTH) was measured in 115 subjects out of total 345 persons, including healthy controls (n = 5), CHB patients (n = 22), and HBV-related cirrhosis patients (n = 88) by chemiluminescent enzyme-labeled immunoassay (ELISA) on the Immulite 1000 autoanalyzer (Immulite 1000, Los Angeles, CA) at the Lawke Health Laboratory Center.

### 2.4 Measurement of serum CYP2R1, CYP27A1, and CYP24A1

ELISA was used to test serum levels of CYP2R1, CYP27A1 (E93538Hu, USCN, US), and CYP24A1 (CSB-EL006401RA, CUSABIO, China) in 94 subjects (10 healthy controls, 28 CHB, and 56 CHB-related cirrhosis). Assays were conducted in accordance with the manufacturer's instructions.

### 2.5 Statistical analysis

Statistical analyses were performed with SPSS 19.0 (IBM, Chicago, IL). For differences between groups, if equal variance was assumed, the significance was assessed by one-way ANOVA analysis for continuous variables. If equal variance was not assumed, the significance was assessed by nonparametric rank sum test. Univariate and multivariate logistic regression analysis was performed to determine the independent parameters associated with vitamin D deficiency (<10 ng/mL). *P* value of <0.05 was considered statistically significant.

## 3 Results

### 3.1 Patient characteristics

**Figure 1:**
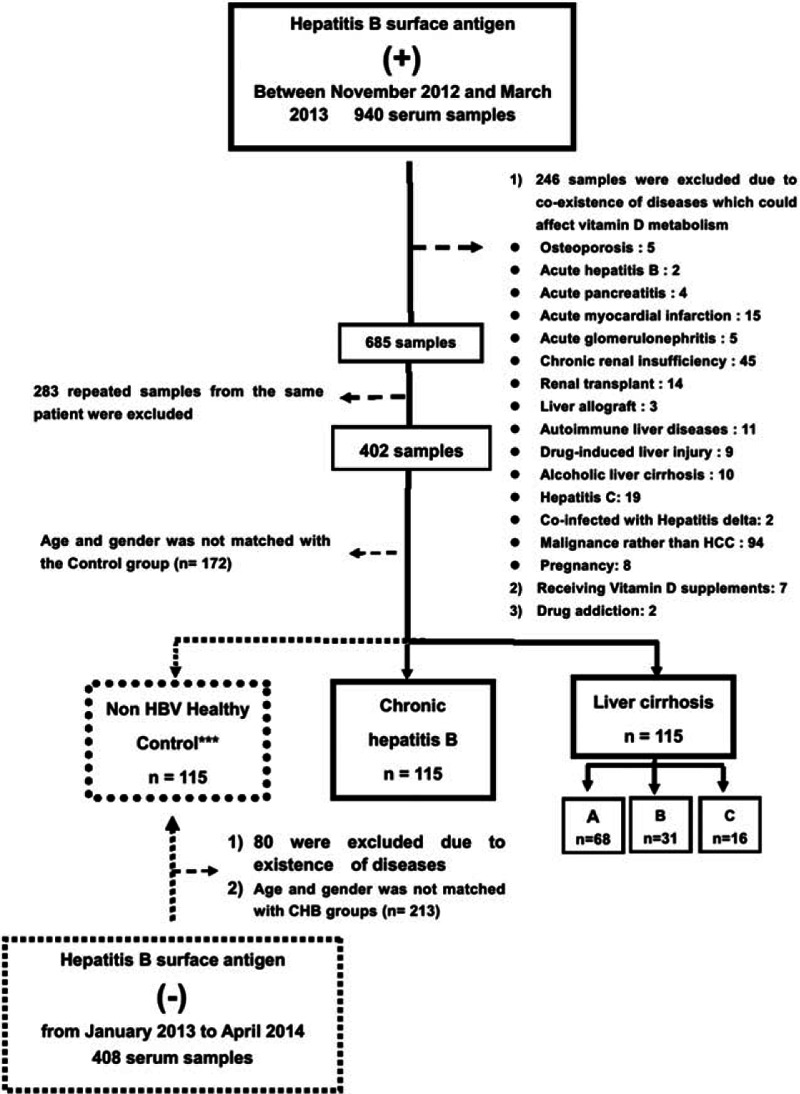
Flow chart for the selection of chronic hepatitis B, cirrhotic patients and non-HBV controls. Out of a total of 940 chronic hepatitis patients, 230 patients qualified and were matched by age and gender. About 115 controls were selected from 408 healthy subjects without HBV infection, who were also matched by age and gender with chronic hepatitis B and cirrhotic patients.

We enrolled 115 eligible persons with chronic HBV infection, 115 cases of HBV-related cirrhosis, and 115 non-HBV healthy controls (age and gender matched with ratio of 1:1:1) into the study (Fig. 1). The HBV-related cirrhosis cohort was further divided into Child–Pugh Class A (n = 68), Class B (n = 31), and Class C (n = 16). There were 84 antiviral treatment-naive patients and 31 CHB patients on nucleos(t)ide analogs. The demographic data, serum biochemistry, minerals, hematological data, HBV-DNA levels, and 25(OH)D3 levels of 345 subjects are summarized in Table 1. In cirrhotic patients, albumin (ALB) levels were significantly decreased (33.95 ± 6.91 g/L), bilirubin (TB) levels were significantly increased (35.41 ± 53.67 μmol/L), and prothrombin time (PT) was significantly prolonged (15.54 ± 3.65 s) when compared with CHB groups (ALB 40.01 ± 4.67 g/L, TB 18.56 ± 26.61 μmol/L, PT 12.36 ± 1.64 s, respectively, *P* < 0.001, Table 1).

**Table 1:**
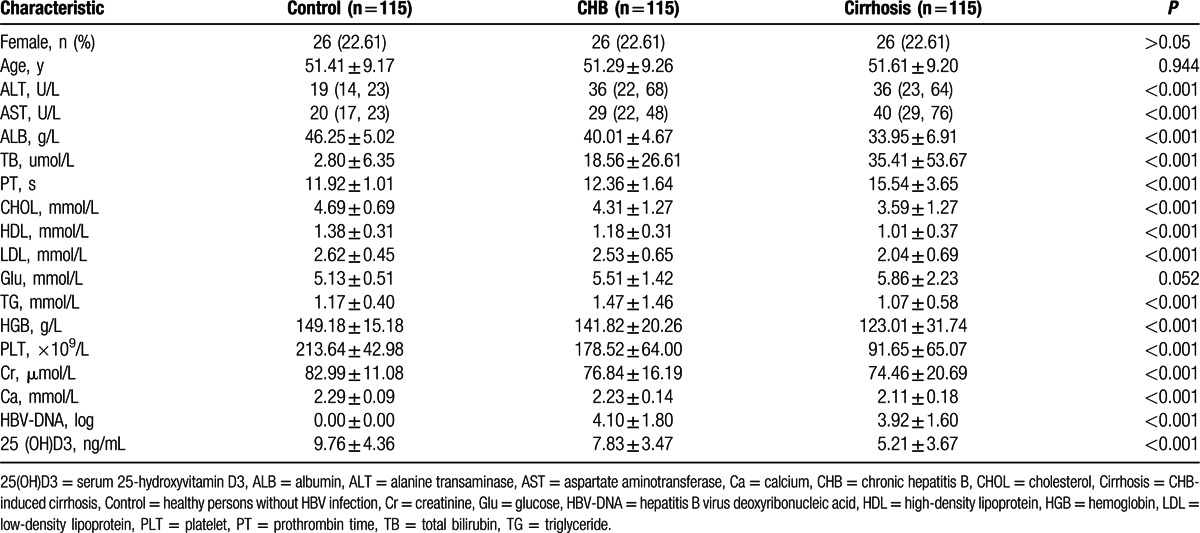
Demographic, biochemical, hematologic, and serum 25(OH)D3 data.

### 3.2 25(OH)D3 serum level decreased with the increase of Child–Pugh Class

**Figure 2:**
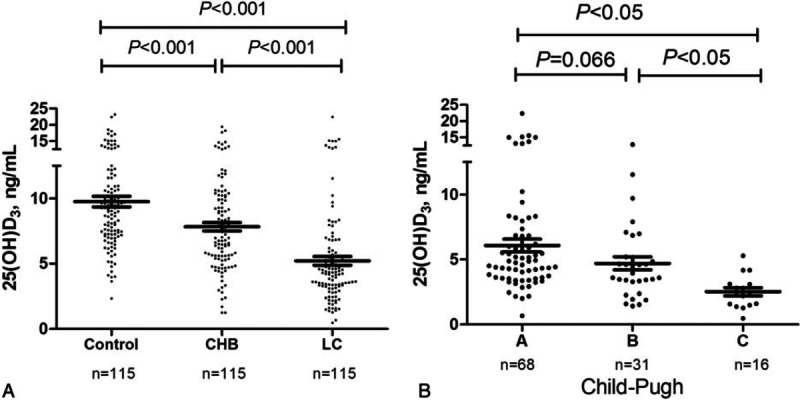
Vitamin D serum concentration significantly decreased with the deteriorated of liver function. Vitamin D serum concentration of CHB patients significantly higher than non-HBV control but significantly lower than that of cirrhotic patients (A). Vitamin D serum concentrations dropped along with Child–Pugh Class in cirrhotic group (B). One-way ANOVA test were used to do the statistical analysis, *P* < 0.05 was treated as significant difference. CHB = chronic hepatitis B, HBV = hepatitis B virus, Control = non-HBV infected healthy control, LC = HBV-related cirrhosis.

There was a significant stepwise decrease in serum 25(OH)D3 levels from healthy controls (9.76 ± 4.36 ng/mL), to persons with CHB infection (7.83 ± 3.47 ng/mL), and hepatitis B related cirrhotic patients (5.21 ± 3.67 ng/mL, *P* < 0.001, Fig. 2A). Of 230 CHB patients, 83.04% of patients had vitamin D deficiency, defined as 25(OH)D3 <10 ng/mL. Furthermore, in patients with cirrhosis, 25(OH)D3 levels significantly decreased along with severity of Child–Pugh class: Class A, 6.21 ± 3.67 ng/mL; Class B, 5.81 ± 2.27 ng/mL; and Class C, 3.21 ± 2.57 ng/mL (*P* < 0.001; Fig. 2B). These results show that vitamin D serum concentration correlates with liver dysfunction.

### 3.3 25(OH)D3 serum levels were not associated with HBV viral loads or antiviral therapy

**Figure 3:**
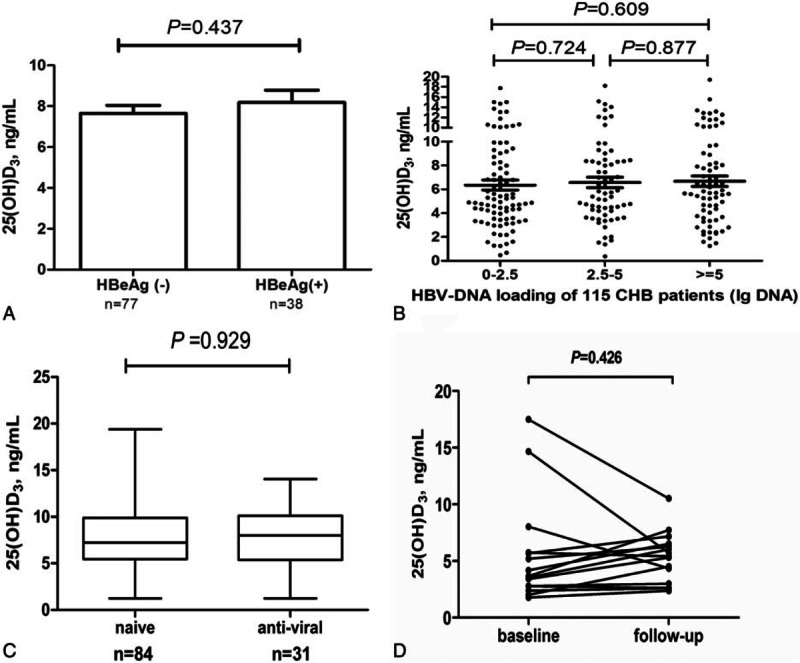
Viral factors were not associated with serum level of vitamin D. No significant difference of vitamin D serum concentrations between HBeAg (+) and HBeAg (–) chronic hepatitis B patients by independent *t* test (A). No significantly difference among patients with high, moderate, and low viral groups by one-way ANOVA test. (B). None significantly difference between patients on antiviral therapy using nucleoside analogs and treatment naïve by independent *t* test (C). In 15 paired patients prior and after antiviral therapy, vitamin D level remains no statistical difference by paired-samples *t* test (D).

There was no significant difference in of 25(OH)D3 levels between HBeAg (+) (8.11 ± 1.67 ng/mL) and HBeAg(–) patients (7.81 ± 2.17 ng/mL, *P* = 0.44; Fig. 3A). Similarly, serum levels of 25(OH)D3 were not different across patients with high (≥5 log IU/L, 6.21 ± 3.67 ng/mL), moderate (2.5–5 log IU/L, 6.24 ± 3.27 ng/mL), and low (≤2.5 log IU/L, 6.31 ± 3.67 ng/mL) viral load (*P* = 0.61, Fig. 3B). Furthermore, 25(OH)D3 was similar between treatment naïve patients (6.21 ± 3.17 ng/mL, n = 84) and patients on antiviral therapy (medium 12 months, range 3–24 months) with nucleoside analogs (7.21 ± 3.67 ng/mL, n = 31, *P* = 0.93, Fig. 3C). We also found that in 15 patients in whom 25 (OH) D_3_ levels were measured prior to and after antiviral therapy with entecavir, there was no statistically significant change in 25(OH)D3 levels (5.55 ± 4.62 ng/mL and 5.34 ± 2.23 ng/mL, respectively; *P* = 0.43, Fig. 3D) despite a virological response (HBVDNA<200 IU/mL). Finally, in cirrhotic group HBVDNA was negative (<200 IU/mL) in 49 patients, between 200 and 10,000 IU/mL in 33 patients and >10,000 IU/mL in 33 patients. Vitamin D serum levels among these 3 groups were not statistically different. These results suggest that vitamin D serum concentration is not significantly altered with HBV viral load or affected by antiviral therapy.

### 3.4 Intact parathyroid hormone, calcium, magnesium, and phosphorus

Of the 345 subjects, intact PTH was measured in 115 (Table 2). In contrast to the change of vitamin D3, there was an upward trend of intact PTH along with the severity of liver disease, although no statistical difference was found. Secondary hyperparathyroidism, defined as intact PTH greater than 69 pg/mL, was found in 3 of 115 cases (2.61%) of CHB patients, but in none of the controls. The mean concentration of calcium was significantly decreased in HBV-related cirrhotic patients (2.10 ± 0.18 mmol/L, *P* < 0.001, Table 2) compared with control (2.23 ± 0.10 mmol/L) and chronic HBV infection group (2.22 ± 0.13 mmol/L). The mean magnesium concentration was significantly lower in chronic HBV infection patients (0.84 ± 0.16 mmol/L) than in controls (1.02 ± 0.11 mmol/L, *P* = 0 .001). However, the levels of magnesium were not statistically different between chronic HBV (0.84 ± 0.16 mmol/L) and HBV-related cirrhotic patients (0.85 ± 0.11 mmol/L). Finally, phosphorus levels were not statistically different among the 3 groups (Table 2).

**Table 2:**
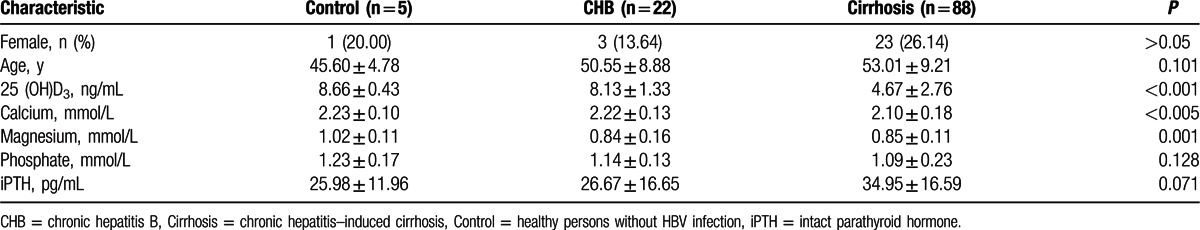
25(OH)D3, iPTH, calcium, phosphate, magnesium in control, chronic HBV infection, and cirrhosis.

### 3.5 Liver dysfunction reserve was associated with vitamin D deficiency

In order to verify independent factors associate with vitamin D deficiency, univariate and multivariate logistic analysis was performed. By univariate analysis, vitamin D deficiency (<10 ng/mL) was significantly associated with higher levels of HBV viral load, AST, TBIL, and prolonged PT, but lower levels of cholesterol, HDL-c, LDL-c, albumin, calcium, and thrombocytes. There is no association between low vitamin D serum level and phosphorus or magnesium. However, multivariate logistic regression analysis showed only higher Child–Pugh Score [with an odds ratio of 1.20, confidence interval (CI): 1.03–1.39, *P* = 0.016] and creatinine level (with an odds ratio of 0.98, CI: 0.96–0.995, *P* = 0.010) independently associated with vitamin D deficiency (Table 3).

**Table 3:**
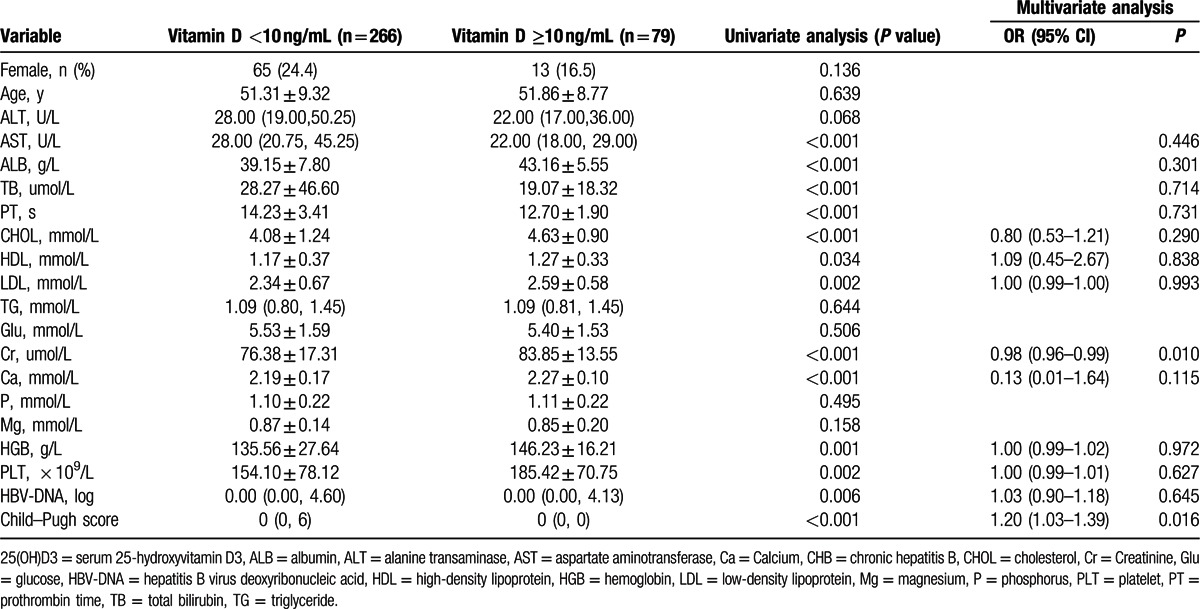
Univariate and multivariate analysis of risk factors associated with severe vitamin D serum level (<10 ng/mL).

### 3.6 Decreased expression of CYP27A1 and increased expression of CYP24A1 was associated with vitamin D deficiency in cirrhotic patients

**Figure 4:**
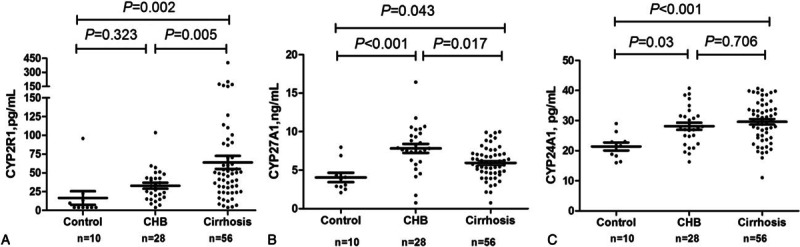
Dysregulation of hydroxylases serum levels relevant to vitamin D metabolism is the possible mechanism of the vitamin D deficiency. Cytochrome P450 cytosomal CYP2R1 for the synthesis of vitamin D, was significantly up regulated (A). In contrast, mitochondrial CYP27A1 the other key synthesizer of vitamin D, was significantly downregulated with the severity of liver dysfunction (B). Degrading CYP24A1 was significantly up regulated (C). One-way ANOVA test was used to do the statistical analysis, *P* < 0.05 was treated as significant difference. Control = non-HBV–infected healthy control, LC = HBV-related cirrhosis.

To study the possible mechanisms whereby vitamin D deficiency worsens with increased severity of liver dysfunction, we measured hydroxylases protein levels of CYP2R1, CYP27A1 (key enzymes in vitamin D synthesis) and CYP24A1 (key enzyme in vitamin D degradation) in 94 out of 345 subjects (Table 4). Serum levels of microsomal CYP2R1 significantly increased in CHB related cirrhotic patients [63.96 (3.92, 403.02 pg/mL)], when compared with non-HBV controls [16.58 (4.00, 95.90 pg/mL, *P* = 0.002)]. However, serum levels of mitochondrial CYP27A1, significantly decreased in cirrhotic patients [5.94 (0.76, 9.97 ng/mL)] when compared with that of CHB patients [7.82, (0.76, 16.45 ng/mL), *P* = 0.017]. The degrading enzyme, CYP24A1, significantly increased in CHB [28.13 (16.31, 40.77 pg/mL), *P* = 0.030] and cirrhotic patients [29.60 (31.45, 11.07, *P* < 0.001)] when compared with controls [21.39 (16.01, 29.01 pg/mL)]. These results suggest that lower levels of CYP27A1 and higher level CYP24A1 together might play an important role in vitamin D deficiency in cirrhotic patients (Fig. 4A–C).

**Table 4:**
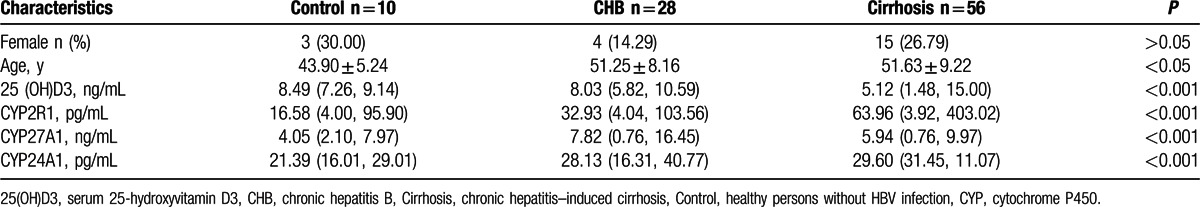
25(OH)D3, CYP27A1, CYP2R1, and CYP24A1.

## 4 Discussion

In this study, we found that vitamin D deficiency (<10 ng/mL) is highly prevalent in persons with chronic HBV infection in northern part of China (84.03%). Our results, together with 2 recent reports from China,^[13,20]^ confirm that vitamin D deficiency in patients with CHB is common living in the northern part of China. More importantly, we found that vitamin D serum level decreased in parallel with the reduced synthetic function of the liver in persons with CHB infection, as confirmed by both univariate and multivariate analysis.

We failed to find any association between viral replication reflected by HBeAg positivity or serum HBV DNA level and vitamin D serum concentration. This is in contrast to what was reported by Farnik et al^[11]^ and Mohamadkhani et al,^[21]^ who found that lower vitamin D was associated with higher HBV replication. This discrepancy may be explained mainly by patients with different disease stages. In their studies, cirrhotic patients only account for a small percentage of total CHB patients. Therefore, the association of vitamin D and liver synthetic function cannot be adequately evaluated. In our study, we recruited a wider disease spectrum of chronic HBV infection, from CHB, to HBV related cirrhosis with different Child–Pugh categories. This broad spectrum of disease allowed us to uncover a relationship between liver function reserve and vitamin D status.^[22–24]^

Furthermore, the impact of seasonal variation on vitamin D status could not be excluded since 1 of these 2 studies did show greater seasonal fluctuation of vitamin D levels than viral levels. To minimize the impact of seasons on vitamin D serum concentration, we only enrolled HBV patients from winter seasons in the north region of China. Additionally, we enrolled 115 healthy volunteers as controls because the normal reference of vitamin D in the Chinese population is not yet established. Recent studies showed that vitamin D in the northern Chinese population is much lower than that in cohorts from the United States or Europe.^[25–28]^ We believe all these measures could strengthen the robustness of our data and our view that vitamin D status is not associated with HBV replication, which is supported by the results of 3 recent studies.^[12–14]^

Indeed, some reports showed that low serum vitamin D concentration does predict adverse outcome of advanced liver diseases.^[5,12,29]^ However, caution should be taken in interpretation of these data since more patients in the eventful group had cirrhosis and the multivariate analysis yielded a statistically significant (*P* = 0.04) yet modest hazard ratio (1.90) with a 95% confidence intervals close to 1 (1.06∼2.43). Therefore, an alternative explanation could be that comprised liver function in cirrhotic patients caused inadequate synthesis and activation of vitamin D. The current cross-sectional study limits us from drawing a firm conclusion on the impact of vitamin D on the natural history of HBV. In order to determine the long-term effect of vitamin D on the progression of CHB, a prospective study of vitamin D supplementation is needed.

The mechanism of vitamin D deficiency in noncholestatic liver diseases is not fully understood. We found that protein levels of mitochondrial CYP27A1 significantly decreased with the deterioration of liver function. This might be explained by failure of production of CYP27A1 due to mitochondrial damage in the scenario of cirrhosis. On the contrary, microsomal CYP2R1, the key enzyme for vitamin D synthesis under normal physiological conditions^[30]^ and may be less affected by liver dysfunction, was upregulated. The latter phenomena may represent compensation in the presence of low serum vitamin D levels. Our results are in accordance with immunohistopathological results of CYP2R1 and CYP27A1 staining on biopsied liver tissues from CHC patients by Barchetta et al^[31]^ and Petta et al.^[32]^ Moreover, CYP24A1, an enzyme for degrading active form of vitamin D, was significantly upregulated in cirrhotic patients. Therefore, the imbalance of these hydroxylases may pay a key role in the pathogenesis of vitamin D deficiency in patients with chronic liver diseases.

In conclusion, vitamin D deficiency is more common in HBV patients than that of non-HBV healthy controls. More importantly, vitamin D serum concentration decreases with higher Child–Pugh Class, which suggests that vitamin D is likely to be a result rather than a cause of impaired liver function and not directly related to hepatitis B viral replication. The pathogenesis of vitamin D deficiency is possibly related to downregulation of synthetic hydroxylase CYP27A1 and upregulation of degrading CYP 24A1 in cirrhotic patients.

## Acknowledgments

We would like to thank Dr Yuanyuan Kong at the Clinical Epidemiology and Evidence Based Medicine Center, Beijing Friendship Hospital, Capital Medical University for her statistical guidance and Dr Peng Jiang at the Lawke Health Laboratory Center, Beijing, China for his technical support. We would like to thank Anthony J. Demetris (MD) and Michelle A. Wood-Trageser (PhD) from University of Pittsburgh Medical Center for language polishing.
